# Pressure Anesthesia

**Published:** 1907-11-15

**Authors:** Charles G. Hampton


### PRESSURE ANESTHESIA.

BY CHARLES G. HAMPTON, D.D.S.

Read before the Detroit Dental Society, May 9, 1907.

Pressure anesthesia is a success in proper hands, and it makes our patients everlasting friends.

I wish to discuss the three forms of application of pressure anesthesia.

Hypodermic, involving the tissues about the teeth. High pressure and also low pressure within the tooth.

Hypodermic Anesthesia. How are we to get at some third molars with the high pressure instrument? In any such case inject hypodermically exactly as for extraction, and proceed to prepare the cavity. If we inject about five drops of ½ of 1% cocain solution lingually and buccally opposite the apex of any tooth in which a cavity is to be prepared, by the time the rubber is in position the main nerve supply is so under the influence that all or most of the pain of excavation is relieved.

In cases that cause the rubber dam clamp to severely crowd the tissues, inject the part before applying clamp. This will relieve much of the pain incident to cavity preparation also.

To anesthetize the lower incisors, cuspids and bicuspids by hypodermic pressure, insert the ordinary hypodermic needle into the tissue on the lingual side of the lower maxilla opposite the mental foramen; that is, between the first and second bicuspids, and anesthetize the main trunks of the nerves leading to these teeth.

Similarly, to effect the superior incisors, cuspids and bicuspids, inject into the gum tissue opposite the infra-orbital foramen, that is, above the cuspid, and anesthetize the main trunks. Of course, this also effects the upper lip, side of the nose and lower eye lid.

*Where pressure anesthesia is not indicated*

No tooth having an exposed pulp, due to the encroachment of decay, should be treated by any form of pressure, whether for anesthesia or any other purpose.

Anesthesia can be readily accomplished in these cases, but we take too great a chance of forcing septic material into dangerous areas. Arsenic is indicated

In cases of chronic or long continued inflammation, or acute hyperemia within the tooth, the contents of the canal is under such unusual blood pressure that it refuses to take up the cocain solution, and only persistent injection will avail, if at all. In such cases relieve the hyperemia by general or local means, then apply pressure anesthesia, or use arsenic if pulp is to be removed.

Some causes of failure in high pressure anesthesia are as follows:

Impurity of water used in cocain solution; impurity of cocain; impurity of other associated drugs; condition of syringe; impurities from the packing of the syringe; lack of care in making *pit* in the enamel and dentine, and countersinking the enamel entrance position of the *pit* relative to the *tubule*; perseverance of operator; immunity of patient; deception, due to nervous patient (not hurt, but looking for trouble, and annoyed by the grinding sounds); leakage of solution from syringe, due to imperfectly fitting washers; improperly shaped syringe point—should be a perfect taper from the point up.

In all cases where possible, employ the rubber dam. In removing pulps insure success by using clean broaches, sterile instruments, proper medication, and allow abundant bleeding.

In the use of the pressure instruments, the application of the principle of hydraulics is reversed. In this case the area of the cross section of the plunger is greater than the area of the outlet. The pressure at the outlet varies directly as the size of the outlet is increased or diminished, provided the pressure on the plunger remains the same. Thus, if 50 pounds pressure is put upon the plunger there will be 50

pounds pressure at the outlet, if the outlet is the same area as the plunger. Reduce the size of the outlet to one-half the size of the plunger, and there will be only 25 pounds at the outlet. Reduce it to one-quarter and there will be only twelve and one-half pounds at the outlet. Reduce the outlet to one-fiftieth the size of the plunger and you will get only one-fiftieth the pressure at the outlet that is exerted upon the plunger. As the latter is about the proportion in the high pressure syringes, there is only one pound pressure at the point for every fifty pounds exerted upon the plunger. In the use of the instrument, there is rarely more than five pounds pressure at the point. More pressure than this may be exerted by the hand method, or so-called low pressure, by using soft rubber.

The injecting pit is one of the stumbling places. In starting the pit use a round burr larger than the syringe point, making a shallow pocket, then with a fissure bur the exact size of the syringe point, very carefully make the pit the exact size of the fissure bur, not allowing the bur to wabble. Then with a cone-shaped bur, slightly taper the inside of the pit, but not taper it all the way down. This will make an absolutely accurate fit, and when carefully prepared saves much annoyance

When drilling the pit take notice of the angle at which you hold the hand piece, and then hold the syringe at the same angle. No other angle will make a perfect fit.

The object of the round bur is to avoid chipping the enamel. The fissure bur assures parallel sides. The cone tapers the wall enough to allow greater contact between the taper point and wall.

*To Obtund for Cavity Preparation Only.*

As the tubuli terminate in Tomes granular layer, and as these granular spaces connect with one another, it is advisable to make the pit within the cavity along the dividing line between the enamel and dentine, which is the position of the granular layer. Warm the entire instrument and save the shock to the tooth of the cold point and cold solution. Bring pressure gently and watch the patient. The solution will be conducted along the granular area, and, as the tubuli terminate in this space, the fluid will be conducted most readily to the pulp chamber along the tubuli. This method will be less rapid than making the pit nearer the pulp chamber, and because the pressure is less direct, there should be less liability of disturbing the tubuli contents or of irritating the nerve. Watch the patient just as closely as in giving a general anesthetic. The eye, the muscular relaxation and respiration will tell us when anesthesia is effective, we should avoid injecting more than is necessary.

We must not expect to be successful in every tooth in the same length of time. A child's tooth contains larger and shorter tubuli and allows penetration more readily. Many teeth are very dense, while others are comparatively porous. We find faulty tubuli due to secondary dentine deposits. Adjacent to old silver fillings, the tubuli may be blocked by metallic oxides. Age varies the length of the tubuli by the deposit of dentine upon the wall of the pulp chamber, thereby increasing the thickness of the wall and diminishing the area of the pulp chamber. Pulp stones occur to hinder penetration.

Dr. Johnson, in making microscopic examination of teeth, discovered cracks extending the entire thickness of the dentine. Some cases in which anesthesia had taken place especially easy, he found the pit had been made in such a crevice, and the solution was conducted thereby directly to the pulp chamber.

If we have the misfortune to make the pit in a position that will not allow penetration, try a new pit and give the instrument a chance to demonstrate its virtues.

I recently had a decidedly stubborn case in a lower first molar buccal cavity, which was *very* sensitive at first, and the patient's temperament was exceedingly nervous. I spent a half hour in one pit applying pressure repeatedly then another half hour similarly in another pit, and the tooth was still tender to the bur. After finishing the operation, my patient told me that her physicians had attempted to place her under both chloroform and ether with little effect, and so much was used that they considered it too dangerous to continue to place her entirely under its influence. Also she said that a cocain solution injected at one time for extraction had little or no effect.

May we consider some persons immune to some or all anesthetics?

Some operators become so skillful in injecting that they can time the period of anesthesia to last until the gold filling is inserted and polished.

In *removing a pulp*, prepare the pit in the same way, locating it in the most available place to point directly at the nerve. After a short application of pressure, follow into the pit with a small bur as far as obtunded, and repeat the application. After the nerve can be exposed, repeat the application of solution gently until no sensation is realized, when the pressure can be increased until all sensation is lost, and then the tooth is ready for pulp extirpation.

### ADRENALINE CHLORIDE.

If adrenaline chloride be added in one to one thousand solution to a cocain solution, it will cause a constriction of the blood vessels, and by thus diminishing the circulation of blood, will allow the cocain to remain in the tissues longer, and prolong the anesthesia.

In injecting for cavity preparation, if adrenaline chloride is used with the cocain the blood vessels of the pulp be, come constricted, not allowing the normal flow of blood-which may cause congestion and destructive action on the pulp.

The time required to clean and shape a cavity after the tooth is rendered painless, is usually not more than twenty minutes. Cocain solution alone in the tooth will hold its effect for a half hour or longer, varying with the amount injected; hence there is no occasion for embarassing the situation within the tooth by unnaturally interfering with the pulps blood circulation and endanger the pulp or pericementum by inviting inflammation. I would advise omitting adrenalin chloride when injecting for cavity preparation.

In removing the pulp the situation differs. By employing adrenaline chloride with the cocain, the blood vessels of the pulp become so constricted that the pulp is completely blanched when removed, and the constriction binds the fibers so closely into a thread-like mass that we have a greater assurance of removing the nerve intact.

The advantage is that only a small per cent, if any, of the solution is conveyed beyond the apex of the root. When the pulp is removed should hemorrhage set in, allow it to bleed freely, and relieve the tissues beyond the apex of all the injected fluid if any has been forced so far. If due care is taken at each step, the canals will be aseptic; but to insure safety carry a mild antiseptic on cotton on a fine broach the entire length of the canals and pack the canal and pulp chamber with DRY cotton for at least a day. The dry cotton is to act as a vehicle to take up blood or other moisture at the time of the hemorrhage when the effect of the adrenaline chloride wears away. Seal the cavity securely with gutta-percha or cement, but not with cotton and sandrac. Do not fill the canals at the same sitting, but allow at least twenty-four hours. Then upon opening we can determine by broaches whether or not any fraction of the nerve remains inside the foramen. If so, it should be removed and the canals enlarged for permanent filling. At the first sitting it would be quite impossible to detect a stump of an anesthetized nerve within the apex, and the possible after-results of leaving it there and packing the canal filling upon it are too annoying to both patient and operator to take the chance.

A successful percentage to use, is:

| | | |
|---|---|---|
| R | Cocaine hydrocholrate | gr. 1-6 |
| | Adrenaline Chloride | gr. 1-1200 |
| | Distilled Water | gtts.3 v |

This gives 3% cocain in the solution. The adrenaline chloride intensifies the cocain action by imprisoning it, so it is not necessary to use as high as 4 or 5 per cent with adrenaline chloride. As low as 1 per cent gives almost as quick and lasting results. Have the tablets on hand and make *fresh* solution each time using. Apply pressure *slowly*, and let the anesthetic enter as a friend, not rush in as a foe.

Now that anesthesia has been successfully effected, the pulp chamber opened and the nerve absolutely removed, is the time the most damage may be inflicted, and all blamed on the pressure process. Some operators will, without washing the hands, after having upon them blood, saliva and septic material from the cavity, twist cotton on a broach between the thumb and fingers and pump vigorously up and down in the canal to remove the blood, and will create many times more pressure at the apex than was given by the pressure instrument, and force more septic material through the apex than could possibly be forced through the tubuli by the pressure instrument. Then they wonder "Why they have pericementitis."

We often find that after applying pressure for, say one minute, the patient will allow us to drill any place in the dentine, or to expose the pulp, but when we use a broach to remove the pulp we find it sensitive and more anesthetic is necessary. Therefore we may conclude that, as the nerves and blood vessels forming the pulp are bound together in an enveloping sheath, the cocain solution passes through the tubuli, and entering the pulp chamber, takes the course of least resistance, which is between the odontoblasts and the encased pulp. This is the area containing the nerve endings; therefore the cocain solution with the least amount of pressure reaches the desired area for cavity preparation.

The opinions of the highest authorities who have given special study to cocain and its action, vary widely in many ways. It is generally conceded that cocain is a protoplasm poison, its effect being due to a chemical reaction, and having a selective action, first for sensory and later for motor nerves.

As the contents of the tubuli is protoplasm, and the contents of cellular structure is protoplasm, we must expect that when cocain is placed in contact with these structures they will be lowered in vitality by the chemical action of the cocain upon them, but not necessarily broken down completely. It is estimated that 1-50 of a drop of 4 per cent. cocain solution entering the pulp chamber will cause complete anesthesia. This small amount or more distributed throughout the pulp chamber and root canal and remaining an hour or two must have only slight breaking down action, because we have so few ill effects. Where ill effects occur close study of the operation will usually show faulty diagnosis, faulty methods or carelessness.

Nature will stand a certain amount of chemical attack or mechanical hard usage and repair the damage; but do not *impose* upon nature.

### TREATMENT.

In case of an exceedingly nervous patient, this formula will prepare them for the operative work:

R Morphine sulphate.................... gr. 1-8
Atrophine sulphate.................. gr. 1-150
M. et. ft. pil No. 1.
Sig.—To be taken one-half hour before the operation.

Or, for the same:

Chloral in dose of 5 to 10 grs., given in water before the operation, for its quieting effect

Or, take antikamnia or ammonol 10 gr. half-hour before operation.

The great majority of cases treated by low or high pressure anesthesia will not be followed by ill effects of any nature, provided the operator has diagnosed the case carefully and has been cautious.

If trouble should arise from any cause, the following treatments are recommended, most of them by Dr. Buckley, of Chicago.

In case of pulp removal when conditions arise which indicate that sepsis is present beyond in the apical region, flood the canals with equal parts of formaline and creosote, and carefully work it to the apex by smooth or twisted broaches and cotton, then seal the cavity securely for the first treatment.

For the second application, if necessary, use 25 per cent formalin and 75 percent creosote. In these conditions same as in treating any abscess, cotton and sandrac for sealing the cavity have little or no place, as they so often increase our difficulties by either allowing the medication to get out or saliva to get in. I find the greatest satisfaction in sealing these temporary medications with cement, because it can be carefully flowed into the cavity without pressure and sets quickly. This avoids the pain which may last several hours if gutta-percha is packed into the cavity and the enclosed solutions crowded into the tissues beyond the apex. It is not necessary to use a thick cement filling—just enough to seal the cavity, then we may rest assured all is safe until we meet the patient again, when the cement may be drilled out in most cases more rapidly than gutta percha can be removed with a heated excavator.

*Tricresol* may be used in place of creosote. It mixes with formalin in all proportions, so that we may vary the percentage of one agent or the other, according to the symptoms. As long as there are indications of putrescent gases within the tooth, stick to the formalin; because the formaldehyde gas unites with the ammonia gas formed by putrescence in the tooth, and they form urotropin—a solid. Also formaldehyde gas unites with the hydrogen sulphid gas generated by sepsis in the tooth, and they form wood alcohol and sulphur. We are thus assured that the gases formed within the tooth will be converted into inocuous solids and liquids, and relieve the pressure so distressing to the patient.

As soon as there are no indications of gas formation, discontinue the *formalin* and use eucalyptol, boracic acid, or any of the effective antiseptics in mild proportions.

If symptoms indicate an abscess arising, a good formula to aid in its relief is:

R Potassium iodide............................ʒ j ss  
Syrup Sarsaparilla Comp....................f ℥ iij  
Sig.—Teaspoonful in water every two hours for three doses, then after meals. This as a laxative, and a stomach tonic..

A saline cathartic is especially valuable to relieve the accumulation of blood in any part, by counter irritation:

R Magnesium citrate.............................℥ j  
Water..............................................ʒ x ij  
Sig.—Take one-half at once and remainder in two hours, if necessary.

If after pressure anesthesia or use of, it sometimes happens that pericemental inflammation follows the use of arsenical nerve pastes, and when the pulp is removed and the root canals are filled, a case of pericementitis results or in case of facial neuralgia, use this formula:

R Tincture aconite...............................f ʒ j  
Chloroform........................................f ʒ iv  
Menthol.............................................gr. xx  
M. Sig.—Saturate a large pledget of cotton and place between gum and cheek opposite effected part.

Again, in case of pericementitis or neuralgia, an excellent formula for internal use is:

R Acetanilid.........................................gr. x  
Simple Syrup.......................................f ʒ ss  
Alcohol..................................q. s. a. d. ℥ jj  
Sig.—Take one-half at once, and remainder in two hours if not relieved.

If a powder is desired for the same purpose, the following serves well:

R Phenacetin.  
Salophen..................................aa. gr. xx  
Make into Powder No. 4.  
Sig.—Take one powder every two or three hours as required.

One operator after employing the high pressure method two years, writes that he has not been able to trace a single case of pathological sequence due to injection. In one case he used the *same* pit three times at 6-minute intervals to obtund for fillings, and the tooth is still in normal sensitive condition.

One well-known dentist writes of between 1500 and 1600 cases of high pressure application in two years and not a single ill effect.

We read of and hear direct from so many who have used pressure anesthesia for two or more years without a single failure, that it gives us heart to continue trying to alleviate pain, without feeling we are treading dangerous ground.

In closing I wish to express the single plea that we continue in our endeavor to master the principles of humanitarian dentistry to the extent our patients will lose that fear and dread, and even the little children will come to us light hearted as to their play room.

### DISCUSSION.

Dr. Giffen. Mr. President and Gentlemen: Dr. Bowles has well said that this is a grand paper. I have had my head full of dental legislation and a few other things for the last few days, so have not given the subject as much thought as I should like to have given it. There are, however, a few points to which I should like to call attention. In the first place, I think the essayist made the statement that pressure anesthesia should never be made to an exposed pulp through decay. I have used cocaine under pressure in just such cases many times. It is a very uncomfortable condition to find when one is treating a tooth that simply has an exposed pulp, that you have produced a septic condition, and possibly the formation of an alveolar abscess, but I think that the cases are very rare where we have a septic condition following pulp extirpation when part of the pulp is healthy. If the pulp is in a gangrenous or septic condition, then of course it is different. I do not know of but one or two cases where septic material has been carried down through a healthy pulp along with the cocaine pressure. I think it is well to use the high pressure syringe for a pulp that we expect to remove, if the cavity is large and we can take in the area through which we drill the pit by enlarging the cavity.

In reference to making a new pit and using arsenic, I prefer to use cocaine pressure.

Another point that I picked up a short time ago from Dr. Graham. I have used cocaine in the usual way on a pledget of cotton, but found trouble many times with saucer-shaped cavities, from the cotton rolling up and allowing the liquid to escape. As to the drilling of the pit: Most of the high pressure syringe men will tell you to use a small burr. Now it is pretty hard sometimes to hold a small bur there long enough to put a cavity through the enamel into the dentine without allowing it to wabble to some extent, and in injecting the pit walls if you have any regurgitation of the liquid, you cannot force it into the dental tubuli. I one day happened to not have a burr in condition to cut enamel that was small enough, so I happened to think of a drill I had—it is the anchor screw drill. It is made for securing the anchor screws, and the bit is possibly 1-8 of an inch longer, with a shoulder, and it will cut in very readily and easily, and there is not so much danger of cutting the walls of your pit untrue. By drilling with this anchor drill and then taking one of the finest Fisher burrs, cone shaped and enlarging the outside, you my set your syringe point into it, and hardly ever miss having a good contact point.

In removing the pulp the essayist spoke about the fragments of pulp tissue being left in the canals. I think many make a mistake by exposing the pulp and passing the broach into it, using a large broach that will lacerate the pulp tissue. We twist the top of the pulp around the broach and pull, and break the pulp off. We should take as fine a broach as we can, pass to the apex of the root if possible, and have it sharp at the point, pass it as cl se to the apical foramen as you can, and then we are not as apt to tear the pulp tissues. When I remove a pulp I generally use, if I think there is any possibility of leaving fragments in, a saturated solution of nitrate of silver with a few fibres of cotton wrapped around a broach, and, after wetting same with silver nitrate, put it loosely up into the canal and let it stay there for a few minutes. In that way you can be sure when you get through that you have got that canal as clear as it can ever be made. The quantity of solution to be used in anesthetizing a pulp of course can only be judged by becoming used to the syringe. It takes but a drop or two, and many of us are apt to make the mistake of using too much as soon as we can get the solution flowing into the pulp chamber, and possibly force some of it through the apical foramen and load the contiguous tissues with the cocaine, which is wrong.

As to adrenilin chloride: It is all right. It is good, but I do not think it has any place in the pulp tissue. It contracts the blood vessels, the arteries and veins, or both, and it forces the cocaine to be retained there. We do not require anesthesia of the pulp as a rule very long, usually a few minutes will do, and we do not wish to have that condition kept up any longer than it is necessary. The cocaine, being protoplasm poison, and the physiological resistance of the pulp tissue being very limited, we are apt to do more damage by leaving it there.

I do not hesitate to fill a root if I know the root canal has been cleaned out, that is, if the live pulp has been removed. There is no danger of septic material being in the canal, and the only reason that Dr. Hampton stated it should not be removed is on account of the anesthetized condition of the apical tissue. I think if you have the canal well opened up it is desirable to use pressure enough to do that.

I have used a high pressure syringe for two years. It took me a good while to decide on the syringe to buy; nearly every syringe had some friends. I finally bought a Sap syringe. I have found out since, from the firm who makes most of them, that the Sap syringe is the best from a mechanical standpoint, the strongest and best made. It is on the screw principle, same as the Higgins. I started in to use the high pressure syringe on a sensitive tooth that I was trying to excavate, and I was so successful that I got enthusiastic, and I think altogether in the two years that I have used it for sensitive dentine on about thirty cases and have not had any unfavorable results at all. At one time I was suspicious as to the pressure that was exerted, and about the result of the action of the cocaine upon the pulp tissue. I now have no hesitation in always using it. I never think of excising a tooth for an abutment, or crowning, or any other method of devitalizing the teeth, but I force the cocaine into the granular layer. It passes then into the dental tubuli; they contain processes of the odontoblastic cells. Suppose the pressure forces the process back and forces those walls out of the way. There is a good deal of pressure exerted, and it appears to me that we have no way of knowing that these cells retained or regained their normal condition. If these processes simply receded and the cocaine diffused through the cell wall producing simply a physiological condition of the pulp that would be all right, or if we knew they would come back again into the dentinal canals, but, it does not seem as though they would. We have made the outer ends of the canaliculi hermetically tight, while the pulpal ends are open or may possibly be filled with secondary dentine, or they may possibly contain some septic matter. The dentine may contain foul gasses or ptomaines which will cause inflammation of the pulp if forced into it.

The physiological resistance of the pulp is not so great in some patients as it is in others, with patients who are suffering from interstitial gingivitis, or from malnutrition, or other diseases, the pulp tissue will not regain its normal condition easily. If it were not for the physiological resistance of the pulp tissue, every time we injected cocaine there would be serious results, and so there must be a certain percentage which will go wrong. I believe when we inject cocaine into the dental tubuli of a tooth to produce anesthesia we are liable to produce a pathological condition of the pulp.

Suppose we have a case of absorption of septic material from the pulp from whatever cause into the apical tissues, and have created an alveolar abscess. We have of course pus with pyogenic organisms. A few drops of pus in a healthy person's system will create here much pain and physical disturbance, and I believe that as soon as we can diagnose pus formation, the tissue involved should be operated. In many cases by opening the canal we relieve the pressure in this way and by using formalin and creosol and such antiseptics, overcome the infectious condition. If we cannot get sufficient drainage through the canal, we must open in the region of the apex of the root. The pus will cause a great deal of trouble if left there. We should not think well of a surgeon who was called, and diagnosed a case of suppuration of pus formation in the palm of the hand, and he would say, "Well, I will give you a sedative, and we will wait a day or two." He would go after the pus if he knew what was the proper thing to do. And when we relieve the pus at a root end we remove the cause of the trouble definitely. While the pus is forming these pus germs, their excretions and toxins are being taken up by the blood, and they affect the nerve centers and finally cause functional inactivity of the vital organs. We must give systemic antiseptics. I use quinine, and believe it is one of the best we have. Besides, we should stimulate the elimination, the salts of magnesium are good for this purpose. If toxic materials are taken up in the circulation to any extent and functional activity is depressed, auto-intoxication follows, and by using a saline cathartic we assist Nature in overcoming this condition.

I think that cocaine or the ether spray are good obtunding agents. Cocaine should be used by cataphoresis. I have two teeth that have been crowned for six years. Dr. Young used cocaine by the cataphoretic method, and I have never experienced the slightest discomfort or any intimation of impending trouble. I presume in some cases this method might injure the pulp. If the cocaine is not forced in by such great pressure, we do not disarrange the physiological function of the tissues, and I think that cataphoresis is preferable. The only objection to it is that it is so slow. Ether spray looks to me to be the most feasible agent that we have today. I have seen it demonstrated two or three times, and I expect to make use of it myself. I have been unable to fill my compressed air tank, so that I have not used it as yet, but it seems to me that if we could eliminate the odor it would be an ideal agent. It will keep the burr cool, as well as the dentine, and prevent the irritation of the heat from the friction. I believe we shall get better satisfaction from it than from the pressure syringe.

Dr. T. R. Buttrick. Dr. Hampton said that where the pulp had been exposed by decay he did not use pressure anesthesia. Cavities in the teeth are usually the result of decay, and it seems to me that you have always got diseased tooth tissue to contend with. I do not like the idea of opening into some other portion of the tooth, and yet I think there must be some danger in drilling into diseased dentine and forcing the contents of the tubules into the pulp. We must open into the tooth quite a distance from the cavity in order to get healthy dentine. I should like to know how Dr. Hampton manages this matter.

Dr. D. N. Graham. I read an editorial in the *Cosmos* that was not very comforting. It suggested that we were doing our patients much harm in catering to a demand for painless operations, and that we are much at fault if we use all kinds of anesthetics. Very soon afterwards I had occasion to have one of my molars filled, and I found that the editor was all wrong. I have used nearly everything that has been suggested in the way of methods for obtunding sensitive dentine: cataphoresis, ether spray, etc., and I think that all have some advantages and also some disadvantages. I was interested in the demonstration with the ether spray in the first case that I tried it on although it was a complete failure. It was, however, an exceptionally difficult case. I think that the ether spray can be of considerable benefit, and can be used to great advantage. It has objections: the most serious is its odor; it is so pungent and volatile that it will fill the office with its disagreeable odor, and the odor of ether to many patients is exceedingly objectionable. The odor of chloroform is also objectionable, and for this reason many patients who require such an obtundant will object very much to the odor of ether or chloroform.

Ether is fast becoming the general anesthetic, it is displacing chloroform very generally, and I fear that if we use it as a spray the objection will be greater than to its systemic administration. It is thought that we can deodorize ether and overcome this objection. Notwithstanding this disadvantage, I think the ether spray is a splendid thing. I have used it in at least a dozen cases. I hope it will succeed in those cases where high pressure and cataphoresis will not, in cases where we have unsuccessfully made an application of arsenic, or when the patient is hyper-aesthetic. In such cases we have the high blood pressure, and I have found cataphoresis almost useless. I have thought the ether spray in these cases would be valuable, but I tried it the other day on one but did not find it successful in that case.

Dr. C. H. Land. I had a case where it was necessary to cut off four perfectly sound incisors; they protruded too far to restore to proper occlusion. An agent came along with a cataphoric outfit, and I gave him a chance to demonstrate. It took him twenty minutes to reduce the sensitiveness in the lateral incisor, and he succeeded very well. I made arrangements that he come back again and try it on the other three teeth in the afternoon. The patient came back in the afternoon and said: "Doctor, how long would it take you to hurt me real bad, and bore right into that tooth and get it out quick?" I said, "About half a minute." She said: "You try it." I said: "It may hurt you pretty bad." She said: "Do so quickly; I do not care."

I drilled into the tooth and incised the crown. "Now finish the others; I do not like to take so much time," said my patient.

I have tried all these things. I have tried this experiment, and find that if a hole is drilled just at the gum line in the dentine it is not painful, if I am careful and take time to go a little at a time, and give the patient a chance to rebound from the shock, the moment I reach the pulp it will hurt a little, but not as much as it will when you go higher up on the tooth. All I do to produce thorough obtunding of the pulp is to pack a little pure carbolic acid on a piece of cotton, and gently drive that straight into the pulp by slight degrees. The pressure alone produces strangulation, and you have no poisonous conditions by a drug. I prefer to use a little drug on the surface, but if I can once get at the pulp I can take it out quickly, and fill immediately. I have practiced dentistry for forty years, and one of the things I first learned was in using arsenic properly. Some say "I would not use it in any tooth." It is because you use it improperly. I have all these years applied it, but never leave it longer than six hours before I remove it. I put it in in the morning, and by four o'clock I can remove part of that pulp. If I cannot, I leave it another twelve hours, and then I can get it out without fail. I have not found it necessary to use many drugs. You only want a few remedies, and nice manipulation. I can take every bit of the enamel off a sensitive tooth, and will not even have my patient say "Oh!" I can demonstrate that at any time, and wish to say that with nice manipulation we do not need all these anesthetics.

Dr. J. J. Travis. I have used high pressure anesthesia for four years this coming summer. I saw a dentist demonstrate high pressure anesthesia at Lansing, at the State Meeting, and when I came home I bought an outfit and used that one a while, and then bought another make. Later on I bought another make, and have been using this continually. I do not use it now as much as I did, but use it when

I think the occasion demands it. I do not know why I don't use it as much as I did. I cannot report anything that would indicate that there is any great danger from using it, even after disintegration of the pulp. We know that little things disturb the circulation in the pulp, yet I cannot report anything that would discourage the high pressure anesthesia where it is really indicated. Perhaps you can hardly call it conservative dentistry, but there are cases where you cannot avoid using it. For extirpation of the pulp, I think it is the finest thing I have ever used, and I use it almost entirely for that purpose.

Dr. M. L. Ward. One thing in particular that has not been spoken of very much, and that has bothered me, is the soreness that is bound to follow the use of the high pressure method for extracting pulps. I have used from 2 per cent to 10 per cent solutions of cocaine, and invariably I find some cases that are tender and lame within twelve hours, which almost precludes any possibility of infection. I had one case in which there were three teeth treated; two of them worked very nicely, but the third was so lame that I could do nothing with it for seven days. I could not tell from what I could observe whether it was simply a natural idiosyncrasy, or whether it was peri-cementitis or infection. It could not have been infection, for it came within twelve hours. We have heard men advocate almost everything for cocaine poisoning, even to the administration of oxygen where the cocaine is supposed to have been taken into the system, and attacked the muscles. If I could tell whether it was peri-cementitis or simply an irritation of the remnants of the pulp left in the canaliculi of the tooth, I might take care of it. If I knew what would counteract the cocaine in those cases, I might take care of that too. One thing that appeals to me particularly in this connection is the too frequent use of cocaine. I am inclined to follow this method: to use cocaine for extracting the pulps in all single-rooted teeth, and arsenic on the first bicuspid and the molars. The root canals of the upper first bicuspid and mesial canals to the upper and lower first molars are difficult of instrumentation. The upper first molar root takes a bend forward, and then backward. Men who have had hundreds of sections made of these teeth find that it is almost impossible to take out these pulps by ordinary instrumentation, or to fill them with chloropercha to the apex of the roots, and they will not recommend the extraction of such pulps by the quick methods. Now, how can you tell that you have extracted all the pulp when you anesthetize by cocaine? Are not the chances better for sloughing it off by arsenic? My judgement is that I am justified in using arsenic in the first bicuspid and first molars. With these exceptions, the soreness and the difficulty of getting to the ends of the roots, I have had very good results. It is only in extreme cases that I use the high pressure syringe for obtunding in extracting a pulp. Of the few that I have observed, there was but one in which there was a particularly bad result. There was considerable soreness of the tooth next day, and it continued for twenty-four hours afterwards. I have never had any soreness from using it in preparing cavities, although I have never used it very much.

Dr. C. H. Land. The only drugs that I have found that has produced soreness, in my practice, have been arsenic, cocaine and formaldehyde, which produced the symptoms Dr. Ward speaks about. One was a case in which I let it stay too long in the tooth.

Dr. T. R. Buttrick. I wonder why the experiment should not be made of drilling a very small hole into the dentine and injecting a small amount of cocaine solution, and then waiting before doing anything in the tooth until the dentine should regain its sensibility, and then drill again into the same hole. If you have forced out the dentinal processes, those individual tubuli would never regain sensitiveness.

I have never used a syringe, so do not know about the pressure; but I would like to know whether this has ever been tried or not. It is the only way that I can see that you can get any definite information.

Dr. Hampton. In regard to Dr. Giffen's speaking of silver nitrate in the root canal, we all know that it is good, but there is a subsequent discoloration that forbids its application, except perhaps in the molars.

Dr. Giffen also spoke of using pressure anesthesia in cases where the nerve has been exposed to the encroachment of decay, and that is something that I wish to impress thoroughly. There are instances in which we can take great care and perhaps be nearly safe, but at the same time we run some risk. When you want to run the least risk in that line, remove as much of the decay as you can by excavating, using as little pressure as possible, then medicate with carbolic acid as thoroughly as you can, and dry out the cavity completely and fill it with enough cement to cover the exposure, and seal the opening; then make a new pit outside the cavity and proceed with the pressure anesthesia: then your pressure is more evenly distributed, as the cocaine enters the pulp chamber, and you do not run the risk of forcing septic material in.

We very often find parts of a tooth which will have to be removed in order to gain access to the filling, especially a gold filling—say the central incisor, we have to get quite good access; but if that cavity is in between so that by drilling right through the part of the enamel and dentine that is going to be removed, good access can be had in that way. Sometimes in occlusial cavities of molars or mesio-occlusial cavities you have to clean out the cavity as best you can and then proceed in a part of the tooth that can be rendered the most nearly aseptic.

Dr. Schaefer asked how to fill the pit. In a place where it may not be removed by the usual cavity preparation, I would enlarge the pit and then fill. If it is to be outside the cavity, and will not be included by the cavity preparation, then of course the position of the pit should be determined by the way we can get at the tooth best and the nature of the filling to be made, or according to the patient who needs the service and as the conditions demand.

I have worked a great deal in the cataphoresis with Dr. Price's apparatus, and have enjoyed the work; but since having the high pressure syringe, I find the cases in which I have used the cataphoretic outfit are so exceptional that I can trace few of them. My batteries are now so entirely broken down from standing that I am not sure the apparatus could be used.

I wish we could have the opportunity of witnessing the removal of enamel and entering into the pulp canals of the teeth as mentioned by Dr. Land, without using drugs. We could avoid the risk of a great many troubles that we must get into with high pressure, if we could use his method successfully.

I wish Dr. Travis had spoken more in regard to the use of cocaine by burnishing it into the dentine. I have not used the process myself, but it sounds practicable.

Dr. Travis. I told Dr. Hampton that in some very sensitive cases where it is not possible to use the high pressure syringe, I have used crystals of cocaine with enough alcohol to moisten slightly in the cavity of the tooth, then take a round burnisher in the engine, and with high speed and constantly increasing pressure, force it into the tubuli enough so that I could cut out a little of it painlessly. In a great many cases the sensitive dentine is only a thin layer. The process can be repeated, cutting off more and more, without affecting the pulp. I have found in labial and crown cavities it has worked very nicely. There are lots of cases where you cannot get at the cavities to burnish well. In some cases it does not work well, where the tubuli are filled with secondary deposits.

